# Effect of Different Opening Diet on the Growth, the Structure of the Digestive Tract and Digestive Enzyme Activity of Larval and Juvenile *Mystus macropterus*

**DOI:** 10.3390/biology13090749

**Published:** 2024-09-23

**Authors:** Xiaoli Li, Xingbing Wu, Xuemei Li, Nian Wei, Ming Jiang, Yongjiu Zhu, Tingbing Zhu

**Affiliations:** Key Laboratory of Freshwater Biodiversity Conservation, Ministry of Agriculture and Rural Affairs, Yangtze River Fisheries Research Institute, Chinese Academy of Fishery Sciences, Wuhan 430223, China; lxl@yfi.ac.cn (X.L.); wxb@yfi.ac.cn (X.W.); xmli@yfi.ac.cn (X.L.); weinian@yfi.ac.cn (N.W.); jiangming@yfi.ac.cn (M.J.)

**Keywords:** *Mystus macropterus*, larvae and juveniles, opening diets, growth, digestive tract morphology and enzyme activity

## Abstract

**Simple Summary:**

*Mystus macropterus*, a commercially significant *Mystus* fish species found in the Yangtze River and Pearl River, is currently experiencing a rapid decline attributed to the construction of hydraulic engineering projects, water pollution, and overfishing. Consequently, extensive artificial breeding efforts are being undertaken for large-scale commercial aquaculture purposes as well as for reintroducing this fish back into its natural habitat. Our aim was the evaluation of the effect of different opening diets on the growth, the structure of the digestive tract, and digestive enzyme activity of larval and juvenile *M. macropterus*. It is recommended to include Tubifex in the diet of *M. macropterus* larvae during the standardized farming process.

**Abstract:**

One of the crucial factors influencing the growth and viability of larvae and juveniles is their opening diets. The objective of this study was to identify suitable initial feed options for *M. macropterus* larvae and juveniles. A total of 1200 newly hatched *M. macropterus* with an average weight of 18.3 mg and an average length of 11.58 mm were selected and randomly divided into four groups. The fish were fed with different opening diets, including rotifer, *Artemia nauplii*, Tubifex, and micro-diet from six days after hatching (dahs), respectively. Growth indices and activities of digestive enzymes were assessed at 10, 15, 20, 25, 30, 35, and 40 dahs. Histological examination of the structure of the digestive tract was performed at 40 dahs, while survival rates were also documented. The results demonstrated that different diets had no effect on the survival rate of larvae and juveniles of *M. macropterus*. The growth performance indices were ranked as follows: Tubifex group > *Artemia nauplii* group > micro-diet group > rotifer group. Remarkably, the Tubifex group exhibited superior growth performance, which was also reflected in the structure of the digestive tract and digestive enzyme activity. Therefore, it is recommended to include Tubifex in the diet of *M. macropterus* larvae and juvenile during the standardized farming process.

## 1. Introduction

*Mystus macropterus*, a medium-sized demersal fish, belongs to the class Osteichthyes, Siluri-formes, Bagridae, and the genus *Mystus*. It is naturally distributed in the Pearl River, Xiangjiang River, Ganjiang River, and Yangtze River systems and commonly inhabits rapids and rocky gravel environments [1]. This fish holds significant economic importance within the *Mystus* genus due to its rapid growth, large size and high nutritional value [2]. However, this species is currently facing a rapid decline due to hydraulic engineering projects’ construction activities, along with water pollution and overfishing. Consequently, extensive artificial breeding programs are being implemented for commercial aquaculture purposes; while also aiming at reintroducing this fish back into its natural habitat. 

Since the 1990s, researchers have been actively involved in investigating the repr0ductive biology and artificial breeding technology of *M. macropterus* [3,4,5], leading to significant advancements in artificial breeding techniques [6]. These developments have greatly facilitated the large-scale breeding and industrialization of *M. macropterus*, thereby accelerating its commercial viability and contributing to its overall growth potential. 

The larva and juvenile stage of *M. macropterus* is defined as the period from 5 to 6 days after membrane emergence, when yolk absorption of larva is basically complete and enters the exo-genetic stage, to the period when various characters and characteristics of the fish are approaching adulthood. The whole stage lasts about one month. *M. macropterus* is a predominantly carnivorous fish species with a diet primarily consisting of animal matter. In its natural habitat, this species mainly preys on benthic invertebrates, such as aquatic insects, snails, clams, shrimp, and crabs [7]. Due to various factors, including environmental conditions and food availability, the survival rate of its larvae is notably low. Despite the fact that artificial breeding techniques for *M. macropterus* have been largely established, low survival rates and suboptimal growth rates of larvae continue to present significant challenges in its aquaculture [8]. The initiation of exogenous feeding during the early developmental stages of *M. macropterus* is crucial for determining the survival and growth rates of the larvae. Specifically, once larvae transition to an exogenous nutrition phase, their survival rate largely depends on the quality and adequate supply of exogenous nutrients [6]. Therefore, selecting nutritionally rich, safe, and suitable starter feeds is a critical factor in ensuring the survival and growth of *M. macropterus* fry.

Various aquatic organisms have been explored as potential feed for fish fry, including tubifex and *Artemia nauplii*. Among these, tubifex have been shown to possess desirable palatability and attractiveness, along with ease of digestion and absorption, leading to improved growth rates and survival rates in tested fry [9,10]. However, Tubifex also present challenges in aquaculture practice, such as the risk of carrying pathogens of aquatic animals, seasonal scarcity, and relatively high prices, which impose limitations on the rearing of juveniles [11]. Similarly, *Artemia nauplii* serve as a feed with good palatability and contribute to higher survival rates among test specimens [12,13]. Additionally, commonly used feeds for newly hatched fish include natural sources such as algae, rotifers, red worms (Tubifex), and nauplii of brine shrimp, as well as synthetic alternatives, such as egg yolk and micro-diets [14].

Currently, the universal method for evaluating opening diets involves comparing the growth rates and survival rates of fry under different conditions. However, there is limited research on the mechanisms that determine the suitability of opening diets, which restricts the further optimization of feed formulations. Factors such as particle size, digestibility, and palatability are important reference indicators for assessing whether a feed can be used for fry [15]. Given that fry are very small, have limited mobility, and have high nutritional requirements for growth, the ease of ingestion and the nutritional content of the feed are also critical factors affecting fry survival and development [16]. Additionally, digestive enzyme activity and digestive tract structure are significant indicators of fish digestive physiology and the utilization of various nutrients [17]. To date, there have been no reports on the ideal opening diet for *M. macropterus*.

Based on the aforementioned research status, this study investigates the larval and juvenile *M. macropterus* as the research subject, using growth as well as the activity of digestive enzymes and the structure of the digestive tract as indicators to evaluate the effectiveness of different opening diets for larvae and juveniles of *M. macropterus*. The aim is to find a suitable opening diet for *M. macropterus* fry, thereby providing a reference for the artificial breeding of *M. macropterus*.

## 2. Materials and Methods

### 2.1. Bait and Experimental Fish

Bait materials, including micro-diet (particle size range: 150–250 μm), *Artemia nauplii* eggs, and Tubifex, were procured from Shandong Shengsuo Fishery Feed Research Center (Yantai, China) and Tianjin Danyang Aquaculture Technology Co., Ltd. (Tianjin, China) Rotifers were collected daily from rearing ponds using a zooplankton net. Table 1 presents the main nutritional components of these materials.

The parental fish of *M. macropterus* were obtained from the Wuhan Yangtze River Fishery Conservation and Research Center of the Chinese Academy of Fishery Sciences, which specializes in domesticating and breeding endemic fish species found in the Yangtze River. The female fish had an average body weight of 269.00 ± 99.52 g, while the male fish weighed around 391.29 ± 68.84 g. The fertilized eggs were obtained through semi-dry fertilization and artificially incubated at a temperature of (27 ± 1) °C with a photoperiod consisting of 14 h of light followed by 10 h of darkness, ensuring dissolved oxygen levels above 6.0 mg/L, ammonia nitrogen content below 0.05 mg/L, and maintaining water pH within the range of 6.5–7.0.

After hatching, the larvae were temporarily placed in a rearing tank with a diameter of 2 m. Following 6 days, the larvae were transferred to three cylindrical rearing tanks measuring 2 m in diameter and 80 cm in height for the experiment. Within each rearing tank, four mesh culture frames with an 80-sized netting were positioned. The frames, made of wooden materials, had dimensions of 50 cm × 50 cm × 10 cm and were securely connected using cable ties. To maintain optimal water quality, the dissolved oxygen level in the rearing tanks was consistently kept above 6.0 mg/L through water inflow devices located at both the bottom and sides of the tanks.

The newly hatched larvae of *M. macropterus* were divided into four groups: rotifer group, *Artemia nauplii* group, Tubifex group, and micro-diet group. Each group had 3 replicates with 100 larvae per replicate. The larvae were placed randomly into the seedling frames. Feeding started from 6 dahs and continued throughout the rearing period with three feedings per day. The feeding times were at 8:00–8:30, 14:00–14:30, and 20:00–20:30. Before each feeding session, any remaining feed and fecal residues in the frames were removed. Daily observations and recordings were made on the behavior and feeding conditions of the experimental fish during the experiment. Since it consists of full feeding, all larval biomass can utilize daily feeding.

### 2.2. Determination of Growth Parameters and Sample Collection 

According to the condition that the larva and juvenile stage of *M. macropterus* lasts for 1 month, the experiment was designed to last for 35 days. The experiment lasted from 30 May 2022 to 15 July 2022. During the experiment, fish mortality rates were recorded daily. The growth index of fish was measured at 5, 10, 15, 20, 25, 30 and 35 days after the start of the experiment. At the same time, three fish in each experimental group were treated with MS-222 (70 mg/L; Sigma, San Jose, CA, USA). After anesthesia, the sample was rapidly decapitated and the caudal fin was rapidly frozen in liquid nitrogen and cryopreserved at −80 °C for determination of digestive enzyme activity. At the end of the experiment (i.e., day 35 after the experiment began), 3 fish were taken from each tank, fixed in Bouin’s solution for 24 h, and then stored in 70% ethanol for digestive histology study.

The length of the fish was measured using a vernier caliper with a precision of 0.01 mm, and the weight of the fish was measured using an analytical balance (precision of 0.0001 g). Thus, the specific growth ratio (*SGR*, %) of the juvenile *M. macropterus* during the experimental period was calculated. The calculation formula is presented below:

*SGR* = [(ln*W*_2_ − ln*W*_1_)/(*t*_2_ − *t*_1_)] × 100%;

where *W*_1_ and *W*_2_ were the weights (g) at times *t*_1_ and *t*_2_, respectively; *L*_1_ and *L*_2_ were the body lengths (cm) at times *t*_1_ and *t*_2_, respectively; *t* was the experimental duration in days; and *n* was the number of individuals in the experiment.

The animal study protocol was approved by the Institutional Review Board (or Ethics Committee) of the Animal Experimental Ethical Inspection of Laboratory Animal Centre, Yangtze River Fisheries Research Institute, Chinese Academy of Fishery Sciences (approval no. YFI2022LXL01) for studies involving animals.

### 2.3. Digestive Tract Sample Measurement

The Bouin’s solution-fixed specimens underwent gradient alcohol dehydration, xylene clearing, paraffin embedding, and sectioning (thickness: 5 μm). Hematoxylin-eosin (HE) staining was performed on the sections followed by mounting with neutral resin. The Nikon Eclipse 80i microscope imaging system (Nikon Corporation, Tokyo, Japan) was utilized for observation and photography while CaseViewer image analysis software (2.4.0.119028) was employed for measurement. Ten randomly selected sections from each group of larval and juvenile fish intestines were analyzed with ten fields of view chosen from each section. Goblet cell density was determined by counting the total number of goblet cells within a range of 100 μm × 100 μm in each field of view.

### 2.4. Enzyme Preparation and Determination of Enzyme Activity

The frozen samples were taken from the −80 °C freezer and thawed on ice. After thawing, the samples were weighed and diluted with pre-chilled homogenization diluent (0.86% sodium chloride solution) in a ratio of weight (g) to volume (mL) of 1:4. The mixture was homogenized. The resulting homogenate was centrifuged at 4 °C and 3000 rpm for 20 min using a low-temperature high-speed centrifuge (Sigma, USA), and the supernatant was collected. After detecting the protein concentration using the BCA method, the pancreatic protease, lipase, and amylase activities in each sample were measured following the operating steps provided in the instructions of the assay kit (Nanjing Jiancheng Bioengineering Company, Nanjing, China) using a full-wavelength spectrophotometer (Thermo, Waltham, MA, USA) or ultraviolet spectrophotometer (UV-2450, Shimadzu Corporation, Kyoto, Japan).

### 2.5. Data Processing and Statistical Analysis

Data were presented as mean ± standard error (mean ± SE). Statistical analyses were performed using SPSS Version 17.0. The total length and weight were analyzed by Two-Way ANOVA followed by Duncan’s multiple comparison; differences were considered significant at *p* < 0.05. Survival rate, specific growth rate, and histological index were analyzed by One-Way ANOVA, followed by Duncan’s multiple comparison; differences were considered significant at *p* < 0.05.

## 3. Results

### 3.1. Effects of Different Opening Diets on Growth and Survival Rate 

**Figure 1:**
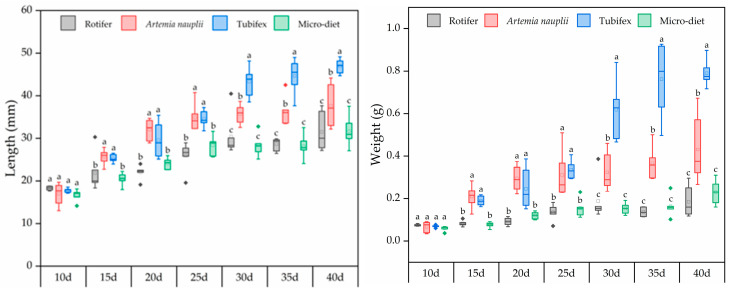
Total length and weight of larval and juvenile *M. macropterus* fed with different opening diets. Note: Different letters abc mean that the length and weight were significantly different under different opening diets at the same time.

The growth and weight gain of larval and juvenile *M. macropterus* in each experimental group are depicted in Figure 1. After being fed with various opening diets, the growth rates of the experimental groups ranked from highest to lowest as follows: Tubifex group (163.56% ± 8.88%) > *Artemia nauplii* group (121.94% ± 26.09%) > micro-diet group (89.96% ± 28.18%) > rotifer group (72.25% ± 26.67%). Similarly, the weight gain rates were observed in the following order: Tubifex group (1038.86% ± 84.17%) > *Artemia nauplii* group (541.41% ± 55.20%) > micro-diet group (288.13% ± 68.93%) > rotifer group (147.84% ± 7.22%).

From 15 dahs until the end of the experiment, the total length of larvae fed with *Artemia nauplii* and Tubifex was significantly greater than those fed with rotifers and micro-diet. Furthermore, from 30 dahs onwards, Tubifex-fed larvae exhibited a significant increase in total length compared to those fed with *Artemia nauplii*. The trend in weight gain was consistent with that of total length. During the same period, groups fed with *Artemia nauplii* and Tubifex had significantly higher weight than those fed with rotifers and micro-diet; additionally, from 30 dahs onwards, Tubifex-fed groups showed a significant increase in weight gain compared to their counterparts who were given *Artemia nauplii*. Statistical analysis using two-way ANOVA revealed that both age in days and feeding ways had a significant impact on body weight and total length (*p* < 0.05), while an interaction between these two factors was also observed (*p* < 0.05) (Table 2), and the most significant interaction occurred at 25–30 d for the Tubifex-fed group (Figure 1).

**Figure 2:**
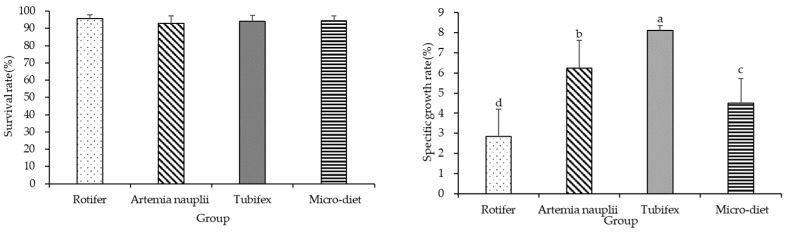
Survival rate and specific growth rate of larval and juvenile *M. macropterus* fed with different opening diets. Note: Different letters abc mean that the specific growth rate was significantly different under different opening diets.

At 40 dahs, there was a significant difference in the specific growth rate (*p* < 0.05) among the groups, with the Tubifex group showing the highest rate (8.11%), followed by the *Artemia nauplii* group (6.25%), the micro-diet group (4.50%), and the rotifer group (2.83%). There was no significant difference in the survival rate of the larval and juvenile *M. macropterus* among the different experimental groups at the end of the experiment, with a range of 93.00–95.67% (Figure 2).

### 3.2. Effects of Different Opening Diets on the Digestive Enzyme Activity 

**Figure 3:**
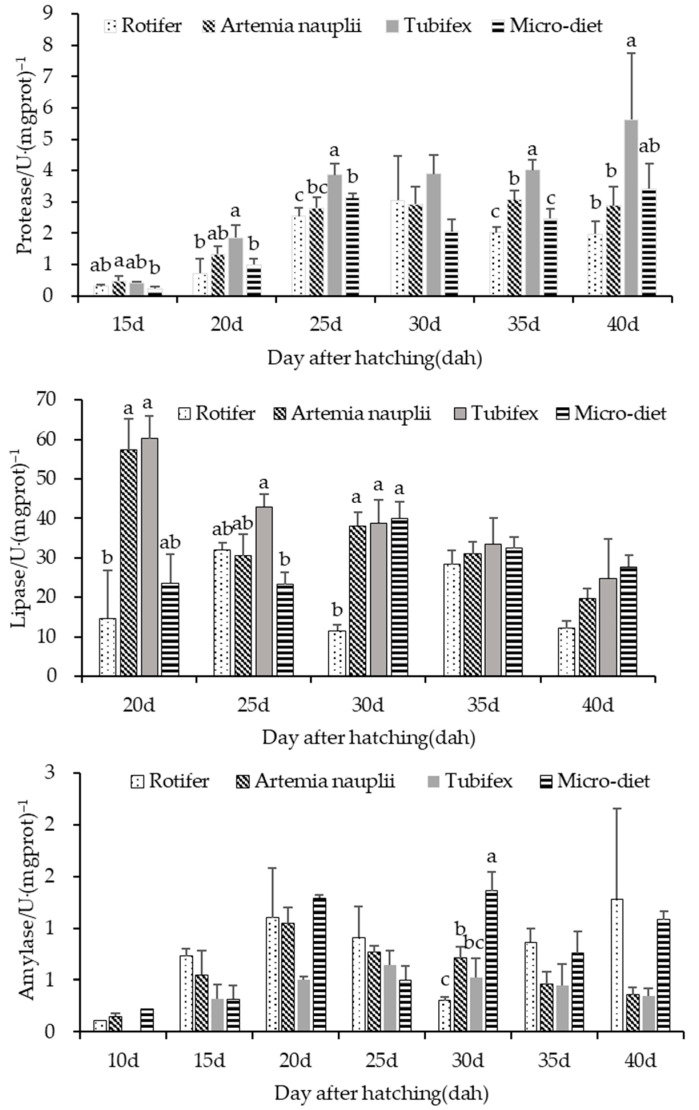
Activities of digestive enzymes of larval and juvenile *M. macropterus* fed with different opening diets. Note: Different letters abc mean that the digestive enzyme activity was significantly different under different opening diets.

The digestive enzyme activities of larval and juvenile *M. macropterus* in different groups are shown in Figure 3. Protease showed a gradual increase from 15 dahs to 40 dahs, with a range of 0.30–5.63 U·gprot)^−1^. The activity of protease in the Tubifex group was significantly higher than that in the other three experimental groups (*p* < 0.05), while the difference between the latter three groups was not significant overall. Lipase activity showed a gradual decrease from 20 dahs to 40 dahs, with a range of 11.58–60.24 U·(gprot)^−1^. The overall activity of lipase in the Tubifex group was significantly higher than that in the *Artemia nauplii*, micro-diet and rotifer groups (*p* < 0.05). The amylase activity showed an increasing trend followed by a decreasing trend in the range of 10 dahs to 40 dahs in the groups of *Artemia nauplii* and Tubifex, while no significant pattern of change was observed in the micro-diet and rotifer groups. The total variation ranged from 0.11 to 1.36 U·(gprot)^−1^. The amylase activity in the micro-diet and rotifer groups was significantly higher than that in the *Artemia nauplii* and Tubifex groups. However, there was no significant difference between the micro-diet and rotifer groups and between the *Artemia nauplii* and Tubifex groups.

### 3.3. Effect of Different Opening Diets on the Structure of the Digestive Tract 

**Figure 4:**
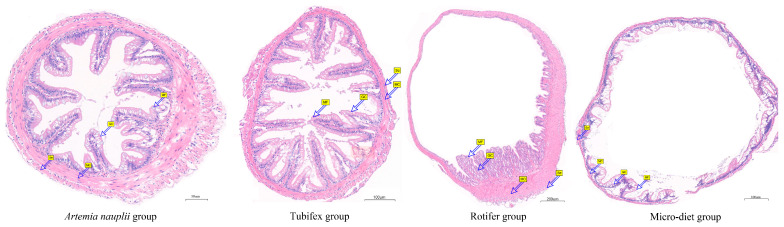
Histological observations of digestive system in larval and juvenile *M. macropterus* fed with different opening diets. GC: goblet cell; MC: muscular; Mu: mucosa; MF: mucosal fold; Se: serosa; SM: submucosa.

The structure of the digestive tract of *M. macropterus* consisted of mucosal layer, sub mucosa, muscular layer, and serosa. The morphological index of the digestive tract tissues is shown in Table 3. The mucosal layer was mainly composed of columnar epithelial cells and goblet cells. The mucosal layer protruded into the lumen, forming mucosal folds of varying heights and sizes. The mucosal epithelium was composed of stratified squamous epithelial cells, with the surface mainly consisting of squamous epithelial cells and goblet cells. The goblet cells were pear-shaped or oval. The submucosa was composed of loose connective tissue, lymphatic vessels, and blood vessels. The muscular layer consisted of longitudinal and circular muscles, but the boundary was not clearly defined (Figure 4). The height of mucosal folds, thickness of muscular layer, thickness of submucosa, and number of goblet cells in the rotifer group were significantly higher compared to the other three experimental groups, while the micro-diet group showed significantly lower values than the other experimental groups. Except for the thickness of the muscular layer, which was significantly higher in the *Artemia nauplii* group than in the Tubifex group, the other three indicators showed no significant differences between the two groups.

## 4. Discussion

### 4.1. Effect of Different Opening Diets on the Survival and Growth of Larval and Juvenile M. macropterus

One of the critical factors influencing larval growth and survival is opening diet. The type, size (palatability), density (availability), nutritional composition, and foraging behavior associated with the opening diet are closely related to the growth and development of larvae [8,15]. In this study, the *M. macropterus* larvae in the Tubifex group exhibited the best growth performance, consistent with their natural foraging behavior, where Tubifex serves as a primary food source [7]. This finding is also observed in studies involving fish species such as *Pampus argenteus* [16]. Furthermore, Tubifex has been shown to have a significant attractant effect, which increases the feeding rate of the larvae [18]. This is likely to be another important reason for the rapid growth observed in *M. macropterus* in the Tubifex group. Although the crude protein content of *Artemia nauplii* is similar to that of Tubifex, the growth performance of *M. macropterus* in the *Artemia nauplii* group was lower compared to the Tubifex group. This difference might be related to the compatibility of feed size with the mouth gape size of the fish [8]. Studies on crab seed feeding also reveal that the small size of *Artemia nauplii* may lead to reduced predation efficiency, failing to meet growth demands [19], which aligns with the results of this study. The interaction between predators and prey (availability) also plays a crucial role in shaping the food preferences of fish larvae [8]. The lower weight gain rate in the rotifer group might be due to the high swimming speed of rotifers, which may be too fast for the *M. macropterus* larvae to capture sufficient food. Throughout the study, *M. macropterus* in the micro-diet group exhibited strong feeding behavior, potentially due to attractive amino acids in the feed [20]. However, the micro-diet group showed the lowest growth indices, likely due to the underdeveloped digestive function of *M. macropterus* larvae, resulting in excessive food accumulation in the gut, which impairs digestion and increases gut load. This inference is supported by the observation of substantial food residue in the gut of the micro-diet group.

### 4.2. The Effect of Different Initial Feeds on Digestive Enzyme Activity in M. macropterus Larvae and Juveniles

The activity of digestive enzymes in fish is closely related to the type and level of nutrients in the feed [21] and the protein composition of the feed [16,21]. In this study, despite similar protein contents among the experimental groups, significant differences in digestive enzyme activity were observed, indicating differences in the protein composition of the four opening diets. Similar findings have been reported in studies involving *Pampus argenteus* [16]. Ali and Jauncey [22] proposed that rapid fish growth leads to increased metabolic rate and protease activity, a phenomenon also documented in studies on juvenile *Takifugu obscurus* and *Mugil cephalus* L. [23,24]. In this experiment, although there were no significant differences in protein content among the different experimental groups, the Tubifex group showed the highest growth indices, with significantly higher protease and lipase activities compared to the other three groups, which is consistent with Ali’s viewpoint.

Studies have shown that the inclusion of artificial feeds can induce changes in digestive enzyme secretion to some extent [18,25]. In this experiment, the amylase activity in the micro-diet group was generally higher than that in the live feed group during the later stages of rearing. This may be attributed to the higher starch and other carbohydrates in the micro-diet, a finding also observed in studies on *Pampus argenteus* larvae [18]. Compared to terrestrial animals, fish have a lower requirement and utilization for carbohydrates, such as starch, particularly in carnivorous species with shorter digestive tracts [25]. In this study, the protease and lipase activities in *M. macropterus* were significantly higher than the amylase activity, indicating that *M. macropterus* is a carnivorous species, consistent with the findings of Zhang et al. (2023) [26]. 

### 4.3. The Influence of Different Opening Diets on the Morphological Index of the Digestive Tract of M. macropterus

During the larval and juvenile stages, digestive tissues and organs, such as the pancreas, liver, and intestine, are not fully developed, and their morphological structures exhibit stress responses to abrupt changes in feeding conditions and nutritional variations [17]. Therefore, maintaining the health and stability of intestinal function and morphology is crucial for the growth of fish. A robust muscular layer of the intestine is believed to promote intestinal contractions, accelerate the absorption of nutrients, and facilitate defecation [27]. In the present study, the morphological differences in the digestive tract tissues can be categorized into two groups: formulated feed and live prey, with the mucosal and muscular layers of the intestine in the live prey group being significantly thicker than those in the formulated feed group. This indicates that the degree of intestinal dilation of the *M. macropterus* in the live prey group is notably higher than that in the formulated feed group, thus aiding in the acquisition of more nutrients. This observation is also noted in Anguilla bicolor bicolor [27].

The well-developed folds in the intestinal wall can increase the absorption surface area of the intestine, ensuring sufficient contact between digestive juices and contents, thereby enhancing nutrient absorption efficiency [28]. The results of this study showed that the mucosal folds in the feed group were dispersed throughout the intestine whereas, in the live prey group, the mucosal folds were dendritically distributed with a significantly greater number of branches than in the feed group, effectively increasing the digestive absorption area of the intestine. This is consistent with what has been found in *Oreochromis niloticus* and *Sarotherodon galilaeus* fingerlings [29]. Intestinal epithelial cells and goblet cells constitute the intestinal surface epithelium, and their secreted products have the functions of lubrication and absorption. HE staining revealed that the number of goblet cells in the single folds of the mucosal layer of the intestine in the live prey group was significantly higher than that in the feed group. This was because fish fed with live prey come into more frequent contact with various external substances, such as microorganisms and suspended particles in the water, which may stimulate the fish’s intestine and trigger a defensive reaction. In contrast, the composition of the artificial formulated feed was relatively simple, causing less intestinal stimulation and thus not inducing an excessive defensive reaction in the fish [30].

Based on these findings, it can be inferred that the intestinal function of *M. macropterus* in the feed group undergoes contraction due to the change in feed, thereby inhibiting the digestion and absorption of nutrients by the intestine. A previous study investigating the impact of commercial feed on juvenile crucian carp *Carassius Carassius* revealed a decrease in hepatocyte and enterocyte proliferation as well as shortened intestinal folds [31]. This aligns with our current study’s results.

Tubifex is highly beneficial for the growth and development of larval and juvenile *M. macropterus*. However, it is necessary to pay attention to the effects of bait on water quality during the actual breeding process, otherwise the water quality may deteriorate. In addition, the conversion method from live bait to compound feed needs further study.

## 5. Conclusions

In conclusion, our findings demonstrate that feeding *M. macropterus* larvae with Tubifex exhibited greater benefits in terms of both survival and growth compared to diets consisting of rotifers, Artemia, or micro-diets. These observations were further supported by analyses of digestive enzyme activity and intestinal structure. Therefore, we recommend incorporating Tubifex into the standardized farming process for optimal larval nutrition in *M. macropterus* cultivation. The study also provided essential data to guide the optimization of the nutritional composition of artificial micro-diet and the development of more suitable micro-diet for this particular fish species.

## Figures and Tables

**Table 1:** Main nutritional components of different opening diets for larval and juvenile *Mystus macropterus*.

Constituents	Micro-Diet (%)	*Artemia nauplii* (%)	Tubifex (%)	Rotifers (%)
Crude protein	52.60	55.25	56.90	53.50
Crude fat	10.30	9.70	9.49	13.23
Crude Ash	12.70	11.16	13.46	/

**Table 2:** Effects of age in days and feeding ways on the total body length and weight of larval and juvenile *M. macropterus*.

	Degree of Freedom	Total Length (mm)	Body Mass (mg)
Feeding methods	3	*F* = 100.818 *p* < 0.05	*F* = 165.298 *p* < 0.05
Age in days	6	*F* = 138.605 *p* < 0.05	*F* = 69.957 *p* < 0.05
Feeding methods × age in days	18	*F* = 7.336 *p* < 0.05	*F* = 16.497 *p* < 0.05

**Table 3:** Morphometrical parameters in digestive tract of larval and juvenile *M. macropterus* fed with different opening diets.

Group	Morphological Index			
	The Height of Mucosal Folds (µm)	The Thickness of Muscular Layer (µm)	The Thickness of Submucosal Layer (µm)	Number of Goblet Cells (Cells/10,000 µm^2^)
Rotifer	203.5 ± 30.14 ^a^	34.14 ± 4.68 ^a^	26.98 ± 3.39 ^a^	4.00 ± 1.03 ^b^
*Artemia nauplii*	111.77 ± 33.05 ^bc^	24.70 ± 3.02 ^b^	8.38 ± 1.22 ^b^	8.80 ± 1.50 ^a^
Tubifex	143.06 ± 14.41 ^b^	12.17 ± 1.47 ^c^	8.30 ± 1.29 ^b^	7.00 ± 1.14 ^ab^
Micro-diet	69.65 ± 7.63 ^c^	8.54 ± 0.65 ^c^	8.26 ± 1.35 ^b^	0.60 ± 0.24 ^c^

Note: Different letters abc mean that the value of morphological index was significantly different under different opening diets.

## Data Availability

The data that support the findings of this study are available from the first author, X.L.
